# Valproate adverse effects on creatine metabolism and transport in a patient under drug therapy

**Published:** 2014-04-03

**Authors:** Fahmi Nasrallah, Joseph Vamecq, Ichraf Kraoua, Marie Joncquel-Chevalier Curt, Moncef Feki, Souheil Omar, Gilbert Briand, Ilhem Turki Ben Youssef, Naziha Kaabachi

**Affiliations:** 1 Department of Biology, School of Medicine, Laboratory of Biochemistry, Rabta Hospital, Jebbari, 1007 Tunis, Tunisia; 2 Department of Biochemistry, School of Medicine, Molecular Biology and Metabolic Diseases, CHRU, 57039 Lille, France; 3 Department of Biochemistry and Molecular Biology, School of Medicine, Laboratory of Biochemistry, Molecular Biology and Metabolic Diseases, CHRU, 57039 Lille, France; 4 Department of Child and Adolescent Neurology, School of Medicine, Mongi Ben Hmida Institute of Neurology, 1700 Tunis, Tunisia; 5 Department of Biology, School of Medicine, Mongi Ben Hmida Institute of Neurology, 1700 Tunis, Tunisia

**Keywords:** Valproate, Creatine, γ-guanidinobutyrate

## Introduction

This manuscript is a report about a patient under valproate therapy an unusual creatine metabolite profile not explainable by known genetic defects primarily affecting creatine biosynthesis and transport. Normal levels of blood ammonia and amino acids, including creatine precursors (glycine and arginine), and the absence of organic acid markers further a priori rule out gene defects secondarily affecting creatine metabolism. In the absence of other etiologic origin, valproate is concluded to cause the abnormal profile combining elevations of urinary creatine and guanidinoacetate in the treated patient. Coherent mechanisms by which valproate may cause the observed metabolite profile are further proposed.

A 16-year-old boy, born to first degree consanguineous parents has no perinatal asphyxia and his had similar illness and she is aged of 14 years. He was presented at the age of 7 years, for psychomotor delay and generalized tonic and clonic seizures partially controlled by valproate (30 mg/kg/day) and clonazepam (0.06 mg/kg/day). Examination showed facial dysmorphism, axial hypotonia with spastic tetraparesis, mental and language retardation and generalized dystonia. Brain MRI showed cerebellar hypoplasia. Auditory evoked potential and electromyography were normal.

Urinary organic acids, ammonemia, and karyotype were normal as well as plasma aminoacids, redox couple showed increased lactate and pyruvate with 5.89 and 0.339 mmol/L, respectively, but the ratio lactate/pyruvate were normal.

**Figure 1:**
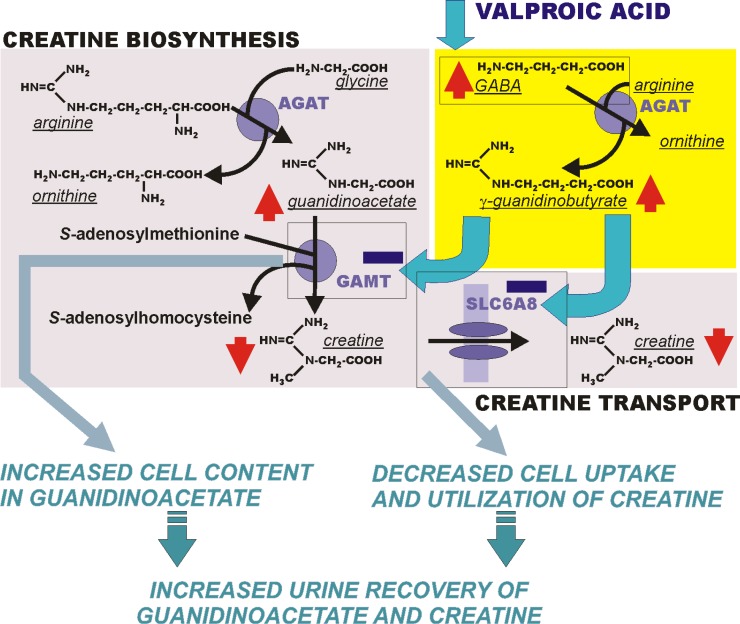
Proposed mechanisms for the valproate adverse effects on creatine metabolism and transport as a coherent cause of the combined increase in urinary guanidinoacetate and creatine.

Urine creatine and guanidinoacetate concentrations were both abnormally elevated (734 µmol/mmol creatinine [normal range: 11–240] and 684 µmol/mmol creatinine [normal range: 4–220], respectively), corroborating disturbed creatine metabolism. This combined metabolite increase was unusual, partially differing from laboratory findings in primary creatine disorders (AGAT, GAMT or SLC6A8 deficiency), excluding increased anapleuresis (arginine, glycine) of creatine biosynthesis. Etiologic search for the rise in guanidino-metabolites pointed toward links between valproate and creatine metabolism previously reported to highlight why exposure to valproate may be a risk for autism development. Valproate causes hyperammonemia and in turn ammonia causes creatine deficiency in the brain, indicating that valproate may lower brain creatine levels.^1^^,^^2^ Besides ammonia-based, a γ-guanidinobutyrate-based link rests on drug-driven increase in brain levels of gamma-aminobutyric acid and resulting availability to yield γ-guanidinobutyrate, an inhibitor of both creatine-forming (guanidinoacetate methyltransferase) and -transporting (SLC6A8) proteins.^3^ This would explain increased recovery of both guanidinoacetate (secondary to cellular inhibition of guanidinoacetate methyltransferase) and creatine (due to its reduced uptake and utilization by cells) in the urine (Figure 1). Concurrence of a SLC6A8 gene mutation, not currently established, might only explain and emphasize the decrease in cell creatine uptake operated by valproate.

In conclusion, association of abnormal concentration of creatine and guanidinoacetate with these clinical features is suggestive of creatine deficiency syndrome, but the combined metabolite increase was unusual and may be related to valproate consumption.
